# Prior Esophagogastric Devascularization Followed by Splenectomy for Liver Cirrhosis with Portal Hypertension: A Modified Laparoscopic Technique

**DOI:** 10.1155/2019/2623749

**Published:** 2019-02-03

**Authors:** Lei Zhang, Hong-Ping Luo, Fei-Long Liu, Wan-Guang Zhang

**Affiliations:** Hepatic Surgery Center, Tongji Hospital, Tongji Medical College, Huazhong University of Science and Technology, Wuhan 430030, China

## Abstract

**Purpose:**

This study was conducted to introduce a novel modified surgical technique for laparoscopic splenectomy and esophagogastric devascularization (LSED) and its safety and efficiency.

**Methods:**

From June 2016 to November 2017, 86 patients were diagnosed with portal hypertension and serious gastroesophageal varices in our center. Of them, 32 patients underwent LSED and 54 received the modified LSED. Results and outcomes were compared retrospectively.

**Results:**

There were no significant differences in preoperative patient characteristics of the two groups. No intraoperative deaths took place in both groups. The intraoperative blood loss was apparently less in the M-LSED group (*P* < 0.05). There was no conversion in the M-LSED group; four patients receiving LSED were converted to hand-assisted LSED due to profuse bleeding during operation (*P* < 0.05). Operation time was significantly shorter in the M-LSED group (*P* < 0.05). Otherwise, postoperative hospital stay was shorter in the M-LSED group (*P* < 0.05). There were no significant differences in postoperative complications between the two groups (*P* > 0.05).

**Conclusions:**

Our study showed that the modified LSED was a safe and effective approach with low conversion rate, less intraoperative bleeding, less blood transfusion, and shorter operation time and postoperative hospital stay compared with classical LSED. Moreover, this technique is relatively easy and technically feasible.

## 1. Introduction

Liver cirrhosis represents the major cause of portal hypertension. Liver failure and bleeding due to esophagogastric varices are responsible for the fatalities in most patients with cirrhosis-related portal hypertension. Endoscopic therapy is a preferred approach for managing esophageal varices, but the endoscopic treatment for gastric varices remains controversial [1, 2]. Open splenectomy and esophagogastric devascularization (OSED), also named as Hassab's operation, is an effective surgical treatment for portal hypertension [3, 4] and can manage hypersplenism and prevent variceal bleeding. However, this technique has not been widely accepted in Western countries because of its high postoperative morbidity and mortality [5, 6].

OSED has a few apparent disadvantages, such as more surgical stress, bigger incision, and more severe postoperative abdominal adhesions that increase the difficulty of ensuing liver transplant surgery [7, 8]. So, a minimally invasive procedure is desirable for this kind of patients. Laparoscopic surgery is a less invasive alternative than open surgery and laparoscopic procedures have improved substantially in terms of skills and technology [9]. In fact, laparoscopic splenectomy and esophagogastric devascularization (LSED) have been effectively and safely employed for the treatment of portal hypertension [10].

Technically, using LSED for cirrhotic patients with portal hypertension and hypersplenism has been a great challenge for surgeons. Massive intractable bleeding, the most severe complication associated with LSED, may necessitate conversion to open surgery. Uncontrollable bleeding tends to occur during the division of the splenic hilar pedicles and the dissection of the upper pole of the spleen [11]. Presented here is a modified LSED (M-LSED), which facilitates the surgical procedure and reduces intraoperative bleeding and conversion to open surgery.

## 2. Patients and Methods

### 2.1. Study Design

This was a retrospective study investigating safety and efficiency of M-LSED. Written informed consents for receiving the surgery were obtained from all the subjects. This study was reviewed and approved by the Institutional Review Board of Tongji Hospital, Tongji Medical College, Huazhong University of Science and Technology.

### 2.2. Patients

From June 2016 to November 2017, 86 patients were diagnosed as having severe esophagogastric varices and/or hypersplenism secondary to portal hypertension at Hepatic Surgery Center of the Tongji Hospital, Tongji Medical College, Huazhong University of Science and Technology. All these patients had a history of refractory variceal bleeding and did not respond to pharmacological and endoscopic treatments. 32 patients received LSED (LSED group), and 54 patients underwent M-LSED (M-LSED group). All operations were performed by the same surgical team.

No patient in our series had received upper abdominal surgery. Preoperative functional hepatic reserve was assessed in accordance with the Child-Pugh scale and all the patients were rated either A or B. Patient's preoperative clinical and laboratory data are shown in Table 1.

### 2.3. Operative Procedures

After induction of general anesthesia, a sandbag was placed under the left shoulder of patient. The laparoscope was introduced through the subumbilical port (12 mm); other three or four ports were used (one 12 mm ports and the others were 5 mm ports). The placement of the trocars depended on patient's body habitus and/or the size of the spleen.

**Figure 1:**
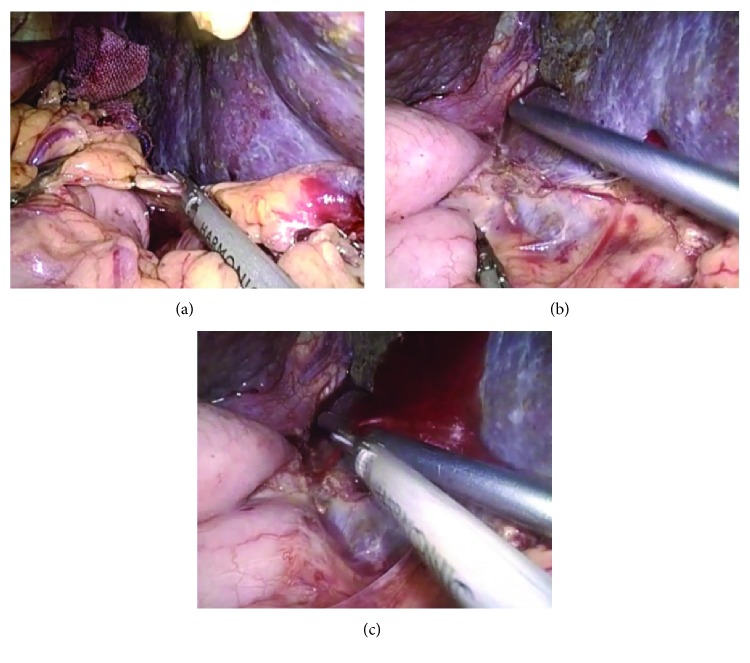
Bleeding from the *vasa brevia*. (a) Division of the splenocolic ligament and gastrosplenic ligament. (b) The *vasa brevia* is too short and not easy to identify. (c) Careless separation of the vasa brevia may cause bleeding from vessels.

Briefly, the LSED went as follows: (1) the splenocolic ligament was divided with a harmonic scalpel (Johnson & Johnson Medical Products, Ethicon Endosurgery, Cincinnati, OH, USA); (2) then, the gastrosplenic ligament, including short gastric vessels, was divided; (3) whenever possible, the splenic artery was identified at the upper border of the pancreas and ligated with nonabsorbable silk suture or a vascular clamp; (4) the splenophrenic and splenorenal ligaments were severed; (5) the spleen was fully mobilized and the splenic artery and vein were transected *en bloc* with a linear cutting stapler (Weck Surgical Instruments, Teleflex Medical, Durham, USA); (6) starting at the middle of the greater curvature of the stomach, devascularization was performed in an inferior-to-superior manner between the gastric serosa and dilated veins. The main branch of the stomach coronary vein was divided with a Hem-o-lok clip; (7) the gastrohepatic ligament was opened and devascularization was conducted along the lesser curvature; (8) the gastric posterior veins were divided; (9) the esophagus was pulled down and the vessels were cut off 5~8 cm above the gastric fundus; (10) in the end, the spleen was removed from the abdominal cavity. LSED is detailed in Figure 1.

**Figure 2:**
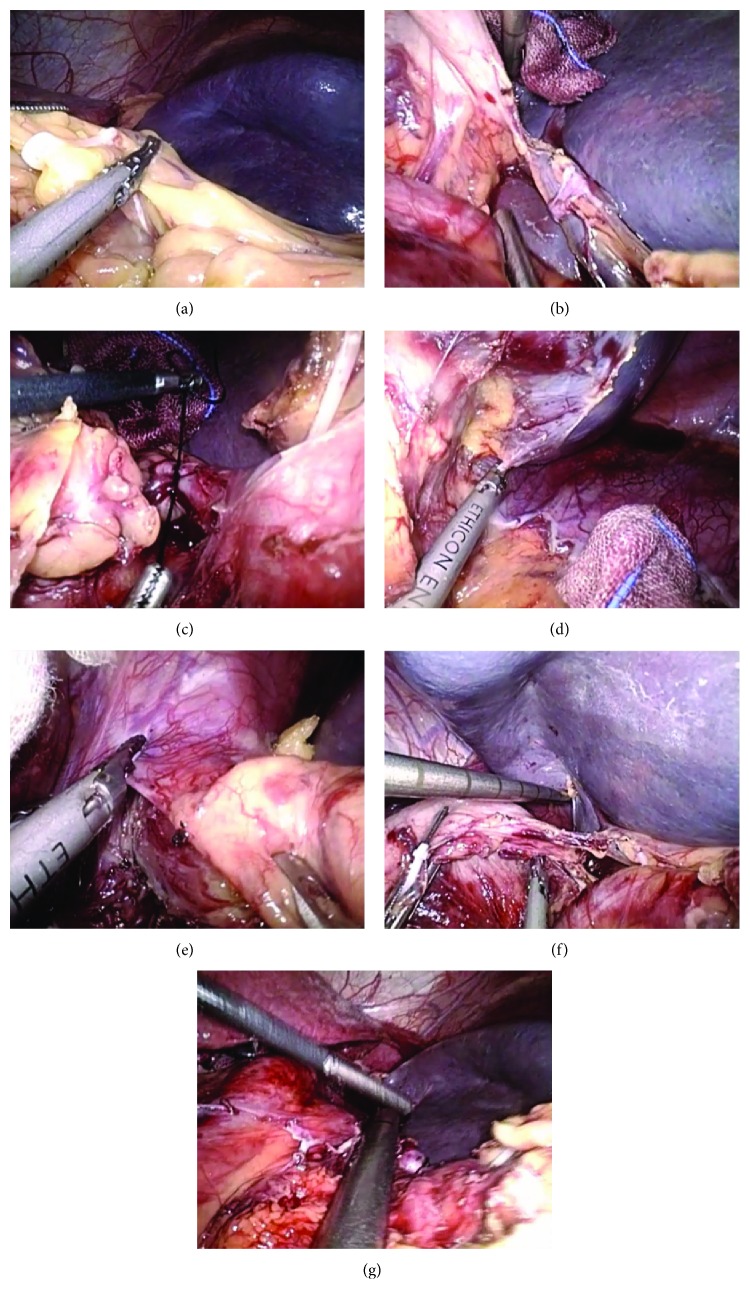
The steps of modified LSED. (a) Division of the splenocolic and gastrosplenic ligaments. (b) The *vasa brevia* is left undivided when it is too short. (c) Ligation of the splenic artery. (d) Division of the splenophrenic and splenorenal ligaments. (e) Esophagogastric devascularization. (f) Division of the short gastric vessels. (g) Transection of the splenic artery and vein.

**Figure 3:**
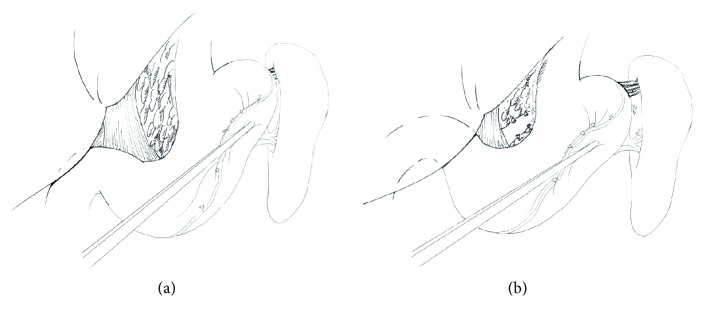
Diagram of modified LSED. (a) The *vasa brevia* is too short and not easy to identify. (b) After devascularization, especially the division of gastric posterior veins, the *vasa brevia* is more obvious and the ligaments looser.

As to M-LSED, the steps were identical to LSED before the ligation of splenic artery. M-LSED differs from LSED in that coronary vein and gastric posterior veins were severed prior to the division of short gastric vessels. The steps of M-LSED were as follows: (1) the placement of the trocars was the same as that of LSED, which depended on patient's body habitus and/or the size of the spleen; (2) the splenocolic ligament was divided with a harmonic scalpel. Bipolar coagulator was used to stanch bleeding; (3) the most important step different from LSED is this step. The gastrosplenic ligament, excluding short gastric vessels, was cut off; (4) the splenic artery was identified at the upper border of the pancreas and ligated with nonabsorbable silk suture; (5) starting at the middle of the greater curvature of the stomach, devascularization was performed in an inferior-to-superior manner between the gastric serosa and dilated veins. The main branch of the stomach coronary vein was divided with a Hem-o-lok clip; (6) the gastrohepatic ligament was opened and devascularization was conducted along the lesser curvature; (7) the gastric posterior veins were divided; (8) the short gastric vessels were carefully identified and severed; (9) the splenophrenic and splenorenal ligaments were divided; (10) the spleen was fully mobilized and the splenic artery and vein were transected *en bloc* with a linear cutting stapler; (11) the spleen was removed from the abdominal cavity. Modified LSED procedure is detailed in Figures 2 and 3.

### 2.4. Statistical Analysis

Statistical analysis was performed by using SPSS 19.0 for Windows. Data were expressed as mean ± standard deviation. A paired Student's *t*-test and Mann-Whitney *U* test were utilized to assess the difference between the two groups. A *P* < 0.05 was considered to be statistically significant.

## 3. Results

All patients had either severe esophagogastric varices (with or without the red color sign) and/or hypersplenism. Preoperatively, all the patients were subjected to liver-protecting protocols and their hepatic function profiles were improved from Child-Pugh class B or C to class A or B. Spleen length ranged from 15 to 45 cm and no significant differences were noted between LSED and M-LSED groups (28.8 ± 7.0 cm vs. 28.0 ± 6.6 cm, respectively; *P* > 0.05). There were no significant differences in other preoperative patient characteristics of the two groups (*P* > 0.05).

Intraoperatively, no deaths took place in the two groups. The mean duration of procedure of the M-LSED group was markedly shorter than that of the LSED group (240.6 ± 39.5 min vs. 184.8 ± 43.6 min, respectively; *P* < 0.01). With four patients in the LSED group, the total laparoscopic LSED had to be converted to hand-assisted LSED due to massive bleeding from the *vasa brevia* (Figure 1). No conversion took place in the M-LSED group, with the difference in conversion rate between the two groups being statistically significant (12.5% vs. 0%; *P* < 0.05). As a consequence, blood loss was less in the M-LSED group than in the LSED group (301.9 ± 75.8 mL vs. 203.3 ± 51.4 mL, *P* < 0.05). Transfusion rate in the M-LSED group was lower than that in the LSED group, but the difference was not statistically significant (34.4% vs. 24.1%, *P* > 0.05). No other major complications were observed intraoperatively.

There were no episodes of postoperative bleeding or encephalopathy. All patients were able to take fluid orally 2 days after the surgery and returned to their normal activities no more than 4 days after the procedure. The M-LSED group had a shorter postoperative hospital stay time than the LSED group (9.7 ± 1.8 d vs. 8.9 ± 1.4 d, *P* < 0.05). The most common complications in the two groups were asymptomatic portal vein thrombosis and temporary ascites; the rates of complications in the two groups were similar (43.8% vs. 38.9%, *P* > 0.05), but the rate of main trunk occlusion of portal veins was zero in both groups. All patients routinely underwent anticoagulant therapy on d2 after operation, a part of patients. The patients with asymptomatic portal vein thrombosis were treated with two dosage of low molecular heparin at least for one month. Other complications included pleural effusion (8 cases) and pulmonitis (4 cases). Examination of discharge tubes revealed that no patients developed pancreatic leakage. The perioperative data are listed in Tables 2 and 3.

A follow-up study, lasting 6 months on average, exhibited that no patient had recurrent variceal bleeding. Endoscopy was performed 1 month after the operation and continued at 3-month intervals. Esophagogastric varices of all patients were relieved during the follow-up period. The mean platelet count rose from preoperative level of (47.4 ± 32.5) × 10^9^/L to (170.7 ± 64.6) × 10^9^/L 6 months after the operation (*P* < 0.05).

## 4. Discussion

Ideally, a procedure for treating portal hypertension should effectively prevent or control hemorrhage, avoid recurrent bleeding from varices, correct severe hypersplenism, exert minor impact on liver functions, and pose minimal morbidity and mortality. Several alternatives are currently available for the treatment of portal hypertension, including pharmacotherapy, liver transplantation, endoscopic sclerotherapy and banding, transjugular intrahepatic portosystemic shunt (TIPS), esophageal transaction, selective or partial shunt, and esophagogastric devascularization. Liver transplantation is generally accepted as the most effective means for treating complicated hepatic cirrhosis and portal hypertension. However, shortage in liver resource and staggering costs restrict the application of transplantation in China. Endoscopic therapy can significantly reduce the rate of rebleeding and mortality, but the treatment alone cannot treat portal hypertension and severe hypersplenism, and long-term studies showed that 10%–30% of patients eventually developed rebleeding. Moreover, endoscopic treatment may lead to serious complications, such as esophageal strictures, ulcers, and even perforation [12]. The efficacy of TIPS, a therapeutic alternative for treating portal hypertension, is similar to that of shunt procedure. But TIPS also has two apparent disadvantages, i.e., causing hepatic dysfunction and encephalopathy, with their rates being approximately 10% and 15%~48%, respectively [13]. The rate of TIPS stenosis and occlusion was reported to vary from 18% to 78%, and they tended to cause clinically significant relapse of variceal hemorrhage and require invasive procedures for reconstitution of flow [14]. Currently, two basic surgical methods are available, i.e., shunt procedure and devascularization. The shunt procedures are more commonly used in Western countries, and devascularization procedures are performed mostly in China and Japan. The shunt procedures can effectively relieve the portal pressure and arrest active variceal bleeding [6]. But this technique is technically complicated, and the incidence of hepatic encephalopathy and mortality was reportedly high [15].

Splenectomy in combination with esophagogastric devascularization can efficaciously resolve the aforementioned problems at the same time. Moreover, splenectomy not only effectively reverses thrombocytopenia but also improves hepatic function, enhances immunity, and ameliorates portal hypertensive gastropathy [16–19]. Though the result of the esophagogastric devascularization has been excellent, this technique has a number of disadvantages, including, among others, more surgical stress, a large surgical incision, high postoperative morbidity, and mortality. Therefore, it is desirable to develop a minimally invasive surgical procedure for the treatment of portal hypertension secondary to liver cirrhosis, which is associated with poor liver function and low tolerance for surgical operation. Laparoscopic surgery is a minimally invasive procedure, and in fact, many minimally invasive procedures have become the gold standards and replaced their open counterparts. Many studies exhibited that LSED was a safe, effective, minimally invasive alternative for treating adult or juvenile portal hypertension [6, 14, 20–23]. Compared with open procedure, LSED was associated with less blood loss and transfusion, faster recovery of gastrointestinal and hepatic functions, shorter postoperative hospital stay, and fewer complications [10, 20–23]. However, cirrhotic patients tend to have massive splenomegaly, advanced collateral vessels, and high risk for bleeding resulting from thrombocytopenia and impaired coagulation and these factors increase the possibility of conversion to open surgery due to massive intraoperative hemorrhage. It was reported that the conversion rate of the procedure varied from 0% to 13% [10].

Massive intractable bleeding is the most severe complication in LSED and tends to occur during the division of the splenic hilar pedicles and during the dissection of the upper pole of the spleen [7, 11, 20, 23, 24]. To avoid the bleeding from splenic hilar pedicles, Poulin et al. embolized the splenic artery before laparoscopic splenectomy [25]. Nicholson et al. performed early hilar devascularization to control the bleeding from the splenic artery and vein [26]. Using the similar techniques reported by other researchers [7, 27, 28], we identified and ligated the splenic artery with nonabsorbable silk suture or a vascular clamp as early as possible. This not only minimized intraoperative blood loss and blood pooling in the extracted specimen but also reduced the size of the spleen, thereby facilitating dissection. According to our experience, the bleeding from the short gastric vessels during LSED was more common than that from the splenic hilar pedicles and difficult to control. In particular, for patients with massive splenomegaly, if the vasa brevia were too short, or dense adhesion existed around the upper role of the spleen, or especially, there was reentry of the upper pole of the massively enlarged spleen, the bleeding from the *vasa brevia* often happened. If the surgeons, especially beginners of laparoscopy, carelessly separate and divide the splenogastric ligament around the major curvature of the stomach, mechanical or thermal injury of the gastric walls may result, in the worst cases, leading to a potentially life-threatening postoperative complication—perforation of the gastric fundus [21, 22]. In this study, we first performed esophagogastric devascularization and then dissected the short gastric vessels. The advantage of this modified procedure lies in the fact that the gastric vessels could be distinctly identified for separation and division because the short gastric vessels would become more obvious and the ligaments looser after division of the gastric posterior veins. Other advantages include less intraoperative bleeding, no conversion, lower transfusion rate, and relatively shorter operation time (since identification of the *vasa brevia* is no longer time-consuming). Another is shorter posthospital stay of the M-LSED group than the LSED group, which was nearly 1 day (*P* < 0.05). Moreover, the M-LSED procedure had similar long-term outcome, compared with those of LSED.

## 5. Conclusions

Our preliminary but encouraging results with the modified LSED demonstrate that the modified procedure is safer and more effective and can, to some extent, prevent massive intraoperative bleeding, lead to low conversion rate, less blood transfusion, and shorter operation time. Moreover, the technique is relatively easy and technically feasible, which may be helpful for newcomers because frequent conversion to open surgery leads to lose their hearts to attempt the LSED procedure.

## Figures and Tables

**Table 1:** Preoperative clinical data for both groups.

Variable	LSED group	M-LSED group	*P* value
Cases	32	54	
Age	46.4 ± 10.2	48.9 ± 12.5	0.315
Sex (M/F)	22/10	38/16	0.874
Child-Pugh class			0.826
A	16	30	
B	14	20	
C	2	4	
Bleeding history	21	38	0.647
Cause of liver cirrhosis (1/2/3/)^a^	18/11/3	36/13/5	0.578
WBC counts (×10^9^/L)	3.3 ± 1.3	2.9 ± 1.2	0.147
Platelet counts (×10^9^/L)	51.6 ± 37.6	45.0 ± 29.1	0.397
Splenic length (cm)	28.8 ± 7.0	28.0 ± 6.6	0.580

^a^Hepatitis B/C/schistosomiasis cirrhosis; WBC: white blood cell.

**Table 2:** Comparison of intraoperative clinical data for both groups.

Variable	LSED group	M-LSED group	*P* value
Cases	32	54	
Operation time (min)	240.6 ± 39.5	184.8 ± 43.6	0.001
Conversion	4/32 (12.5%)	0/54	0.017
Blood loss (mL)	301.9 ± 75.8	203.3 ± 51.4	0.001
Transfusion	11/32 (34.4%)	13/54 (24.1%)	0.303
HAL	4/32 (12.5%)	0/54	0.017

HAL: hand-assisted LSED.

**Table 3:** Comparison of postoperative data for both groups.

Variable	LSED group	M-LSED group	*P* value
Cases	32	54	
Postoperative hospital stay (days)	9.7 ± 1.8	8.9 ± 1.4	0.025
Complications			0.874
Pulmonary effusion	3	5	
Pulmonitis	2	2	
Pancreatic leakage	0	0	
Postoperative bleeding	0	0	
Main trunk thrombosis in portal vein	5	8	
Encephalopathy	0	0	
Temporary ascites	3	6	
Total	13 (43.8%)	21 (38.9%)	

## Data Availability

The database used for statistical analysis that provided data used to support the findings of this study are restricted by the Hospital Ethical Board in order to protect patient privacy. Data are available for researchers who meet the criteria for access to confidential data. For more information, researchers may contact Zhang WG, MD, (wgzhuang@medmail.com.cn).
